# NEDD9 promotes cancer stemness by recruiting myeloid-derived suppressor cells ***via*** CXCL8 in esophageal squamous cell carcinoma

**DOI:** 10.20892/j.issn.2095-3941.2020.0290

**Published:** 2021-08-15

**Authors:** Dongli Yue, Shasha Liu, Tengfei Zhang, Yong Wang, Guohui Qin, Xinfeng Chen, Huanyu Zhang, Dong Wang, Lan Huang, Feng Wang, Liping Wang, Song Zhao, Yi Zhang

**Affiliations:** 1 Biotherapy Center, the First Affiliated Hospital of Zhengzhou University, Zhengzhou 450052, China; 2 Cancer Center, the First Affiliated Hospital of Zhengzhou University, Zhengzhou 450052, China; 3 Department of Etiology and Carcinogenesis and State Key Laboratory of Molecular Oncology, National Cancer Center/Cancer Hospital, Chinese Academy of Medical Sciences (CAMS) & Peking Union Medical College (PUMC), Beijing 100730, China; 4 Biomed Innovation Center, Yehoo Group, Shenzhen 518067, China; 5 Department of Thoracic Surgery, the First Affiliated Hospital of Zhengzhou University, Zhengzhou 450052, China; 6 School of Life Sciences, Zhengzhou University, Zhengzhou 450052, China; 7 Henan Key Laboratory for Tumor Immunology and Biotherapy, Zhengzhou 450052, China

**Keywords:** Esophageal squamous cell carcinoma (ESCC), cancer stem cells (CSCs), neural precursor cell expressed, developmentally downregulated 9 (NEDD9), myeloid derived suppressor cells (MDSCs), C-X-C motif chemokine ligand 8 (CXCL8)

## Abstract

**Objective:**

Esophageal squamous cell carcinoma (ESCC) has high morbidity and mortality rates worldwide. Cancer stem cells (CSCs) may cause tumor initiation, metastasis, and recurrence and are also responsible for chemotherapy and radiotherapy failures. Myeloid-derived suppressor cells (MDSCs), in contrast, are known to be involved in mediating immunosuppression. Here, we aimed to investigate the mechanisms of interaction of CSCs and MDSCs in the tumor microenvironment.

**Methods:**

ESCC tissues and cell lines were evaluated. Neural precursor cell expressed, developmentally downregulated 9 (NEDD9) was knocked down and overexpressed by lentiviral transfection. Quantitative PCR, Western blot, immunohistochemistry, cell invasion, flow cytometry, cell sorting, multiplex chemokine profiling, and tumor growth analyses were performed.

**Results:**

Microarray analysis revealed 10 upregulated genes in esophageal CSCs. Only NEDD9 was upregulated in CSCs using the sphere-forming method. NEDD9 expression was correlated with tumor invasion (*P* = 0.0218), differentiation (*P* = 0.0153), and poor prognosis (*P* = 0.0373). Additionally, NEDD9 was required to maintain the stem-like phenotype. Screening of chemokine expression in ESCC cells with NEDD9 overexpression and knockdown showed that NEDD9 regulated C-X-C motif chemokine ligand 8 (CXCL8) expression *via* the ERK pathway. CXCL8 mediated the recruitment of MDSCs induced by NEDD9 *in vitro* and *in vivo*. MDSCs promoted the stemness of ESCC cells through NEDD9 *via* the Notch pathway.

**Conclusions:**

As a marker of ESCC, NEDD9 maintained the stemness of ESCC cells and regulated CXCL8 through the ERK pathway to recruit MDSCs into the tumor, suggesting NEDD9 as a therapeutic target and novel prognostic marker for ESCC.

## Introduction

Esophageal cancer is one of the most common fatal cancers and sixth most common cause of cancer-related mortality worldwide^1^. Esophageal squamous cell carcinoma (ESCC) is the major histopathological subtype of esophageal cancer. Despite the development of multimodality therapies, the prognoses of patients remain poor. Thus, further investigation is necessary to obtain insight into the mechanism of therapeutic resistance and tumorigenesis of this malignant cancer.

Increasing evidence has shown that cancer stem cells (CSCs) possess features of self-renewal, multilineage differentiation, and resistance to conventional cancer therapy, which are the driving forces of tumorigenesis and metastases^2^. The CSC concept was first described by Dick and co-workers^3^, who showed that tumorigenic properties were attributed to a minority population of leukemia cells, which were identified based on their expression of certain surface markers to distinguish them from non-tumorigenic cells. During the last decade, CSCs have been detected in many types of solid tumors, and their phenotypic and functional characteristics have been widely investigated^4–6^. As CSCs are thought to be the main cause of therapeutic failure, these cells are considered as potential therapeutic targets.

Numerous studies have indicated that immune cells in the tumor microenvironment play a prominent role in tumor progression and metastasis. Myeloid-derived suppressor cells (MDSCs), a heterogeneous population of myeloid cells that include monocytic (M-MDSCs) and granulocytic (G-MDSCs) cells, are among the most important players mediating immunosuppression. The immunosuppressive effects of MDSCs are relatively well-studied in patients with cancer^7–9^. Recent studies have shown that MDSCs endow cancer cells with stem-like properties^10^. This study was therefore conducted to determine the mechanisms of interaction between CSCs and MDSCs in the tumor microenvironment, and provide a basis for developing more specific therapeutic targets.

## Materials and methods

### Patients and tumor samples

A total of 135 ESCC tissues and 90 neighboring non-cancerous tissues samples were freshly obtained from 51 females and 84 males diagnosed from January 2012 to December 2013 at the Department of Thoracic Surgery of the First Affiliated Hospital of Zhengzhou University. The mean age of the patients was 62.52 years (40–84 years). The clinicopathological characteristics of patients with ESCC are summarized in **Supplementary Table S1**. The collection of samples was approved by the Ethics Committee of the First Affiliated Hospital of Zhengzhou University (approval number: 2018-KY-92) and informed consent was obtained from each patient.

### Cell lines and cell culture

The ESCC cell lines, KYSE70, KYSE150, KYSE450, KYSE510, and EC9706, were preserved in our laboratory and maintained as a monolayer in RMPI 1640 (Hyclone, Logan, UT, USA) supplemented with 10% fetal bovine serum (FBS; Gemini Bio, West Sacramento, CA, USA), 100 IU/mL of penicillin, and 100 µg/mL of streptomycin at 37 °C in a humidified 5% CO_2_ incubator (Thermo Fisher Scientific, Waltham, MA, USA).

### Analyzing and sorting side populations of cells using FACS

ESCC cells were analyzed by fluorescence-activated cell sorting (FACS) when the cells were in the logarithmic growth phase. The cells were digested with 0.025% trypsin and 1 mM EDTA, washed with calcium/magnesium-free phosphate-buffered saline (PBS), and then resuspended in DMEM (supplemented with 2% FBS) at a concentration of 1 × 10^6^ cells/mL, followed by incubation at 37 °C in 5% CO_2_ for 10 min. Following incubation, the DNA-binding dye, Hoechst 33342 (Sigma-Aldrich, St. Louis, MO, USA) was added at a final concentration of 7.5 µg/mL either independently or in the presence of 100 µmol/L verapamil (Merck, Kenilworth, NJ, USA) and incubated for 90 min in the dark with intermittent mixing. The cells were then washed with ice-cold PBS and filtered through a 40-µm cell strainer to obtain single-suspension cells. Five minutes before analysis and sorting of the cells by FACS (FACS Diva Option; BD Biosciences, Franklin Lakes, NJ, USA), a final concentration of 1 µg/mL propidium iodide (PI, Sigma-Aldrich) was added. Hoechst 33342 was excited with a UV laser at 350 nm and fluorescence emission was measured with 405/BP309 (Hoechst blue) and 570/BP20 (Hoechst red) optical filters. PI labeling was measured through a 630/BP30 filter to detect dead cells. PI-negative cells were sorted into two subpopulations, Hoechst 33342-negative cells (SP cells), whose ability of fluorescent efflux could be blocked by verapamil, and Hoechst 33342-positive cells (non-SP cells). Finally, the two groups were collected for sorting, purity evaluation, and further experiments.

### Microarray analysis

Total RNA was extracted from tumor cells using TRIzol reagent (Invitrogen, Carlsbad, CA, USA) according to the manufacturer’s instructions. Microarray analysis was performed following a standard protocol (Capital Bio, Beijing, China). Fluorescently-labeled (Cy5 and Cy3-dCTP) cDNA was prepared using Eberwien’s linear RNA amplification method and a subsequent enzymatic reaction. To measure technical replicates, a one swap-dye experiment was performed on each biological sample. Arrays were scanned with a confocal LuxScan™ scanner and the images obtained were analyzed using LuxScan™ 3.0 software (both from Capital Bio). Space- and intensity-dependent normalization based on the locally weighted scatterplot smoothing (LOWESS) program was performed.

### Sphere formation assay

To investigate the ability of the cells to form cell spheres, 1,000 cells were seeded into 24-well Ultra Low Attachment Plates (Corning, Corning, NY, USA) in serum-free DMEM/F12 medium supplemented with heparin, B27, epidermal growth factor, and basic fibroblast growth factor. After culturing for 7 days, the number of spheres was counted using a microscope (Leica, Wetzlar, Germany). Three independent experiments were conducted in parallel.

### RNA extraction and the quantitative real-time polymerase chain reaction (qRT-PCR)

Total RNA was extracted from ESCC cell lines and tissue specimen by TRIzol reagent (Invitrogen), and first-strand cDNA was synthesized from 1 µg of total RNA using the PrimeScript RT reagent Kit with gDNA Eraser (TaKaRa, Shiga, Japan) as described previously^11^. The cDNA was used as a template for qRT-PCR, which was performed using the FastStart Essential DNA Green Master mix (Roche, Basel, Switzerland) and assessed with an Agilent Mx3005P (Agilent Technologies, Santa Clara, CA, USA). *GAPDH* was used as an internal control. All reactions were performed in triplicate. The data were analyzed using the 2^ΔΔCt^ method. The sequences of primers used for qRT-PCR are shown in **Table 1**.

**Table 1:** Primer sequences for all genes tested

Gene	Sense sequence	Anti-sense sequence	Product size
*GAPDH*	GCACCGTCAAGGCTGAGAAC	TGGTGAAGACGCCAGTGGA	138 bp
*NEDD9*	GACCGTCATAGAGCAGAACAC	TGCATGGGACCAATCAGAAGC	114 bp
*OCT4*	GGGAGATTGATAACTGGTGTGTT	GTGTATATCCCAGGGTGATCCTC	144 bp
*NANOG*	CAAAGGCAAACAACCCACTT	TCTGCTGGAGGCTGAGGTAT	158 bp
*SOX2*	TGAGCGCCCTGCAGTACAA	GCTGCGAGTAGGACATGCTGTAG	84 bp
*LIN28*	CGGGCATCTGTAAGTGGTTC	CAGACCCTTGGCTGACTTCT	191 bp
*KLF4*	ACACAAAGAGTTCCCATCTCAAG	GGTAGTGCCTGGTCAGTTCATC	123 bp
*ALDH1A3*	GCACCGACTATGGACTCACA	AGGGCGTTGTAGCAGTTGAT	112 bp
*CXCL8*	GTTGTAGGGTTGCCAGATGC	TTCTCCCGTGCAATATCTAGG	190 bp
*CD33*	GCCCCAGGACTACTCACTC	CCAGCGAACTTCACCTGACA	91 bp
*CD271*	CCTACGGCTACTACCAGGATG	CACACGGTGTTCTGCTTGT	109 bp
*CD90*	ATCGCTCTCCTGCTAACAGTC	CTCGTACTGGATGGGTGAACT	135 bp
*NOTCH1*	GAGGCGTGGCAGACTATGC	CTTGTACTCCGTCAGCGTGA	140 bp
*NOTCH2*	CCTTCCACTGTGAGTGTCTGA	AGGTAGCATCATTCTGGCAGG	96 bp
*HES1*	TCAACACGACACCGGATAAAC	GCCGCGAGCTATCTTTCTTCA	153 bp
*HES3*	GCACGCATCAATGTGTCACTG	CCTTCTCCAATTTGCGCTTCC	88 bp
*WNT3a*	AGCTACCCGATCTGGTGGTC	CAAACTCGATGTCCTCGCTAC	430 bp

### Immunohistochemistry

The paraffin-embedded ESCC tissues and their paired adjacent non-cancerous tissues were examined for the expression of Neural precursor cell expressed, developmentally downregulated 9 (NEDD9) using anti-NEDD9 antibody (1:200, Abcam, Cambridge, UK). Sections were treated with xylene and rehydrated through a series of decreasing alcohol concentrations. Antigen retrieval was performed by boiling the sections in 0.1 M citrate buffer. Endogenous peroxidase activity was suppressed by incubation in 3% hydrogen peroxide for 30 min. The sections were incubated overnight with primary antibodies at 4 °C. After incubation with horseradish peroxidase-conjugated secondary antibody for 1 h at 37 °C, the sections were treated with the substrate, 3, 3′-diaminobenzidine, counterstained with hematoxylin, and visualized using a microscope (Leica). Detailed evaluation of immunohistochemical staining experiments was performed as described previously^12^. The high NEDD9 expression group had scores equal to or exceeding the median level of expression, whereas the low NEDD9 group had scores less than the medium level of NEDD9 expression.

### The Transwell migration and invasion assay

Migration and invasion assays were performed using Transwell chambers as described previously^13^. The cells (2 × 10^4^) were plated onto the top chamber with a non-coated membrane (for the migration assay) (24-well insert; 8-µm pore size; Corning) or with a Matrigel-coated membrane (for the invasion assay) (24-well insert; 8-µm pore size; BD Biosciences). After 24 h of incubation at 37 °C, the cells that did not migrate or invade through the pores were removed with a cotton-tipped swab. Cells on the lower surface of the membrane were fixed with 4% paraformaldehyde, stained with 0.5% Crystal Violet, and counted using a microscope (Leica) at 200× magnification in 10 random fields. Both experiments were independently repeated three times.

### Lentiviral generation and cell sorting

The lentiviral GV248-shNEDD9, pBpLV-NEDD9, and corresponding control vectors were purchased from GenePharma (Shanghai, China). All inserted sequences were confirmed by DNA sequencing. To obtain a stable cell line overexpressing NEDD9, we infected KYSE70 cells with lentiviruses containing pBpLV-NEDD9. To acquire a stable cell line with NEDD9 knockdown, we infected KYSE450 cells with lentiviruses containing GV248-shNEDD9. After 48 h, the transfected cells were sorted by MoFlo XDP (Beckman, Brea, CA, USA) based on the expression of green fluorescent protein. The expression of NEDD9 was confirmed by RT-PCR and Western blot.

### Western blot

Total proteins were extracted in RIPA lysis buffer (Beyotime, Shanghai, China). The protein concentration was determined using an Enhanced BCA Protein Assay Kit (Beyotime). The protein samples were denatured by boiling for 10 min. Equal amounts of proteins were loaded onto a 10% SDS-PAGE gel for electrophoresis and then blotted onto a polyvinylidene fluoride membrane (Millipore, Billerica, MA, USA). The membrane was blocked in 5% nonfat milk and incubated with primary antibodies overnight at 4 °C, followed by treatment with secondary antibody for 2 h at 37 °C. Primary antibodies used were as follows: anti-NEDD9, anti-pSTAT3 (Tyr705, 1:1,000), anti-pNF-κB (Ser536, 1:1,000), anti-pERK1/2 (Thr202/Tyr204, 1:1,000), and anti-β-actin antibodies (1:1,000; Cell Signaling Technology, Danvers, MA, USA). Signals were detected by enhanced chemiluminescence detection reagents (Pierce, Rockford, IL, USA). Three independent experiments were performed for each analysis, and the gels were run under the same experimental conditions.

### Flow cytometric analysis

The cells were collected and dissociated into single cells suspended in PBS containing 3% FBS. The cells were stained with fluorescence-conjugated monoclonal antibodies against CD271 and CD90 (BD Biosciences) for 20 min in the dark at 4 °C. The corresponding isotype immunoglobulins were used as controls. Dead cells were identified using 7-AAD (Biolegend, San Diego, CA, USA). Data were acquired on a BD FACS Canto II cytometer (BD Biosciences) and analyzed with Diva analysis software (BD Biosciences). Three independent experiments were performed for each analysis.

### Multiplex chemokine profiling

Human multiplex chemokine detection was performed using a LEGENDplex™ Multi-Analyte Flow Assay Kit (Biolegend), which is a bead-based immunoassay for simultaneous quantification of multiple analytes in a single sample using a standard flow cytometer, according to the manufacturer’s instructions. Cell culture supernatant samples were collected, the testing reagents (assay buffer, mixed beads, and detection antibodies) were added, and samples were shaken for 2 h in the dark at room temperature. Streptavidin-phycoerythrin was subsequently added, which bound the biotinylated detection antibodies, resulting in fluorescent signal intensities proportional to the amounts of bound analytes. Samples were washed once and resuspended in wash buffer. The data were read on a flow cytometer and quantified with LEGENDplex™ software. Samples were evaluated in duplicate.

### Cell isolation and sorting

ESCC specimens were cut into small pieces and digested in RPMI 1640 (Gibco, Grand Island, NY, USA) supplemented with 0.25% trypsin (Gibco), 0.002% DNaseI (Gibco), and 20% FBS (Gibco) at 37 °C for 20 min. Tumor-infiltrating leukocytes within dissociated cells were filtered through a 100-µm mesh and isolated by Ficoll-Hypaque density gradient centrifugation (Beijing Chemical Reagent Company, Beijing, China). The mononuclear cells were washed and resuspended in medium supplemented with 10% heat-inactivated FBS for FACS analysis. The PBMCs (peripheral blood mononuclear cells) were sequentially isolated by Ficoll-Hypaque density gradient centrifugation (Beijing Chemical Reagent Company) using the anti-CD14 and anti-CD11b MACS magnetic sorting system (Miltenyi Biotech, Gladbach Bergisch, Germany) within 2 h of sample collection. According to the manufacturer’s instructions, PMN-MDSCs (CD14-CD11b+) and M-MDSCs (CD14+CD11b+) were enriched. The purity of the two subsets of cells was > 95%.

### Mouse experiments

All the xenograft mouse model experiments were conducted in the Henan Key Laboratory for Pharmacology of liver diseases, and the animal protocol was approved by the ethics committee of the Henan Key Laboratory for Pharmacology of liver diseases (approval number: 2019-41). Female BALB/c-nu mice (15–18 g, 5–6-weeks-old) were obtained from the animal facility (Beijing Vital River Laboratory Animal Technology, Beijing, China). The mice were divided randomly into groups. ESCC cells (1 × 10^6^) were injected subcutaneously into the left flank of recipient BALB/c-nu mice (5 mice in each group). Tumor-bearing mice were treated with Reparixin (20 µg/mouse, once daily intraperitoneally from day 7 to day 14), G-MDSCs (1 × 10^7^/mouse, intravenously on day 14), or a combination of both. The control mice received vehicle alone. Tumor growth was monitored using a caliper, and the tumor volume was calculated according to the formula: (length × width × width)/2. The animal facility was pathogen-free and was maintained under a 12-h light-dark cycle (room temperature: 20–24 °C; humidity: 40%–60%) conditions. The animals had free access to feed and water. At the end of the experiments, all mice were sacrificed by CO_2_ inhalation.

### Statistical analysis

Data are expressed as the mean ± standard deviation. The Student’s *t*-test was used to analyze continuous variables with a normal distribution between 2 groups, and one-way analysis of variance was used to compare 3 or more groups. The paired *t*-test was used to analyze paired samples. Pearson’s χ^2^ and Fisher’s exact tests were used to analyze categorical variables. Spearman’s rank correlation analysis was used to study the relationships between variables. The Kaplan-Meier method was used to determine the overall survival curve. The Cox proportional hazard model was used for multivariate analyses. Statistical analyses were conducted with SPSS statistical software for Windows, version 17.0 (SPSS, Chicago, IL, USA). All tests were two-tailed, and *P* < 0.05 was considered as statistically significant.

## Results

### NEDD9 expression was enriched in stem-like cells and involved in ESCC progression

**Figure 1:**
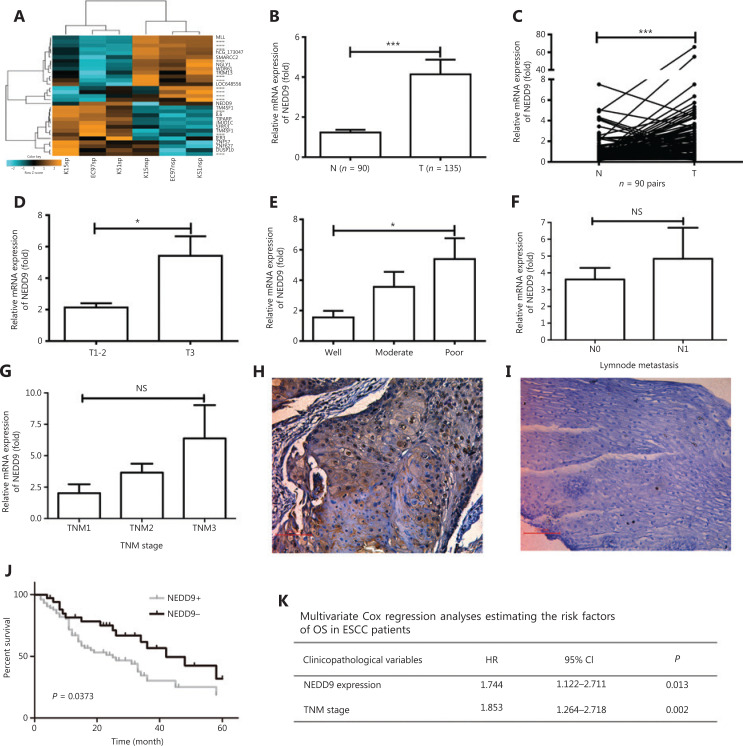
NEDD9 expression is higher in stem-like cells and plays a role in esophageal squamous cell carcinoma (ESCC) progression. (A) Microarray analysis was performed to identify changes in gene expressions in side population (SP) and non-side population (NSP) cells. (B) NEDD9 expression in neighboring non-cancerous tissues (N, *n* = 90) and ESCC tissues (T, *n* = 135) was determined by real-time PCR. (C) NEDD9 expression in 90 representative carcinoma tissues (T) and their corresponding non-cancerous tissues (N) from the same patients were analyzed for comparison. The correlation of NEDD9 expression with clinicopathological characteristics such as tumor invasion (T1-2: *n* = 58; T3: *n* = 77; D), differentiation (well: *n* = 45; moderate: *n* = 68; poor: *n* = 22; E), lymph node metastasis (N0: *n* = 98; N1: *n* = 37; F) and TNM stage (I: *n* = 10; II: *N* = 96; III: *n* = 29; G) were analyzed. Immunohistochemical staining of NEDD9 in primary ESCC samples (H) and their corresponding non-cancerous tissues (I). (J) Kaplan-Meier curves for overall survival rate of ESCC patients according to expression status of NEDD9 (NEDD9 high expression short as NEDD9+: *n* = 79; NEDD9 low expression short as NEDD9−: *n* = 56). (K) Multivariate analyses of overall survival using the Cox’s proportional hazard model. *P < 0.05; ***P < 0.001.

Side population (SP) cells and tumor sphere-forming culture assays are the main methods used to isolate CSCs without the requirement for cell-specific markers^14,15^. To identify genes associated with cancer stemness, we used mRNA microarray analysis to compare the mRNA expression profiles of pairs of SP and non-SP cells in ESCC cell lines (EC9706, KYSE150, and KYSE510). The results identified 10 upregulated mRNAs (*NEDD9, TM4SF1, IL6, TIPARP, JMJD1C, SFRS3, IER3, ZNF57, ZNF627, and DUSP10*) in SP cells compared to those in the non-SP cells (**Figure 1A**), indicating that the significantly altered expression of these genes was involved in the stem-like phenotype of ESCCs. We used qRT-PCR to validate the expression of the 10 genes in tumor sphere-forming stem-like cells, when compared to the parent cells. Consistent with the results obtained in SP cells, we found that only NEDD9 was upregulated in tumor sphere-forming stem-like cells, when compared to the expression in parent cells (**Supplementary Figure S1**). These data suggested that NEDD9 is a candidate CSC biomarker of ESCC.

Next, the mRNA expression of *NEDD9* in ESCC tissues *vs*. that in neighboring non-cancerous tissues was examined. The results revealed frequent upregulation of *NEDD9* in ESCCs compared to adjacent non-cancerous tissues (**Figure 1B and 1C**). Moreover, *NEDD9* expression was significantly correlated with tumor invasion and differentiation, but not with tumor-node-metastasis (TNM) stage or lymph metastasis (**Figure 1D–1G**). Next, we performed immunohistochemistry analysis of the paraffin-embedded human ESCC tissues and found that NEDD9 protein expression was significantly upregulated in ESCCs, when compared to that in adjacent non-cancerous tissues (**Figure 1H and 1I**). As shown in **Figure 1**, *NEDD9* expression was significantly correlated with tumor invasion and differentiation, but not with other variables such as age, sex, TNM stage, or lymph node metastasis. Furthermore, Kaplan-Meier analysis showed that overall survival was significantly shorter in patients with NEDD9 overexpression (**Figure 1J**). Multivariate analysis showed that NEDD9 expression (*P* = 0.046) and TNM stage (*P* = 0.003) were independent prognostic factors (**Figure 1K**). Together, these results suggested that NEDD9 played a key role in the tumorigenesis and progression of ESCC.

### NEDD9 was required to maintain a stem-like phenotype

**Figure 2:**
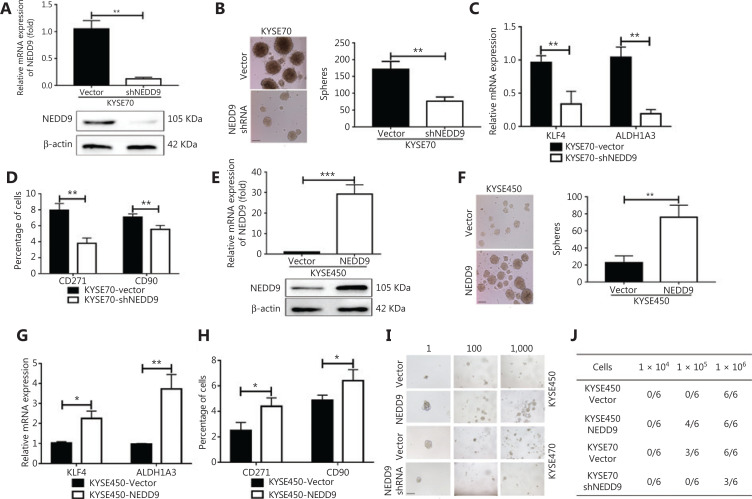
NEDD9 is required for maintenance of a stem-like phenotype. (A, E) Expression of NEDD9 was confirmed between NEDD9 knockdown/overexpression and vector cells. (B, F) The capability of tumor sphere formation was measured between NEDD9 knockdown/overexpression and vector cells. Scale bar = 100 μm. (C, G) Expression of stemness-related transcription factors, KLF4 and ALDH1A3, was detected between NEDD9 knockdown/overexpression and vector cells. (D, H) Expression of stemness markers, CD90 and CD271, was analyzed by flow cytometry between NEDD9 knockdown/overexpression and vector cells. (I) Cells were dissociated into 1, 100, and 1,000 cells for 7 days of suspension culture. Scale bar = 100 μm. (J) Tumorigenic cell frequency in each fraction of ESCC cells was determined by a limiting dilution assay in NOD/SCID mice. *P < 0.05; **P < 0.01; ***P < 0.001.

To determine the functional significance of the preferential expression of NEDD9, we examined the effect of NEDD9 downregulation and overexpression on the maintenance of CSCs in ESCC. We first confirmed NEDD9 expression in human esophageal cancer cell lines (KYSE70, KYSE150, KYSE450, KYSE510, and EC9706). NEDD9 expression was highest in KYSE70 and lowest in KYSE450 (**Supplementary Figure S2**). Targeting NEDD9 in KYSE70 cells by lentiviral-mediated shRNA significantly reduced the mRNA and protein levels of NEDD9 (**Figure 2A**). NEDD9 knockdown severely impaired tumor sphere formation, an *in vitro* indicator of self-renewal and proliferation capacities (**Figure 2B**). CSCs are marked and regulated by various stemness-related transcription factors, including KLF4^16,17^, and ALDH1A3^6,18,19^, both of which are essential for CSC maintenance. NEDD9 knockdown significantly decreased the expressions of KLF4 and ALDH1A3 (**Figure 2C**). Expressions of the CSC markers, CD271 and CD90, which are widely used ESCC stemness-related markers, was shown to be correlated with the tumor-initiating capacity of CSCs^20–22^. The percentages of CD271^+^ and CD90^+^ cells were also dramatically reduced by NEDD9 shRNA (**Figure 2D**). We next evaluated the effects of NEDD9 overexpression on stemness-associated phenotypes in ESCC cells. NEDD9 expression was confirmed by qRT-PCR and Western blot (**Figure 2E**). NEDD9 overexpression significantly enhanced tumor sphere formation (**Figure 2F**), and also elevated the expression of stemness-related transcription factors, including KLF4 and ALDH1A3 (**Figure 2G**). The percentage of CD271^+^ and CD90^+^ cells was also increased by NEDD9 overexpression (**Figure 2H**). We further performed a limited dilution assay both *in vitro* and *in vivo* to investigate stemness regulated by NEDD9. For the *in vitro* limited dilution assay, we found that in the KYSE70-shNEDD9 group, the size and numbers of spheres were significantly decreased compared to that of the KYSE70-shNC group, whereas overexpression of NEDD9 in KYSE450 cells increased the size and numbers of spheres compared to the effects of the KYSE450-vector (**Figure 2I**). For the *in vivo* limited dilution assay, we subcutaneously injected 10^6^, 10^5^, and 10^6^ KYSE70-shNC, KYSE70-shNEDD9, KYSE450-vector, and KYSE450-NEDD9 overexpressed cells into BALB/c-nude mice. The results showed that NEDD9 knockdown reduced the tumor incidence, whereas NEDD9 overexpression in KYSE450 cells increased the tumor incidence (**Figure 2J**). These results showed that NEDD9 played a critical role in maintaining stem-like malignant properties.

### Targeting NEDD9 suppressed tumor growth *in vivo*

**Figure 3:**
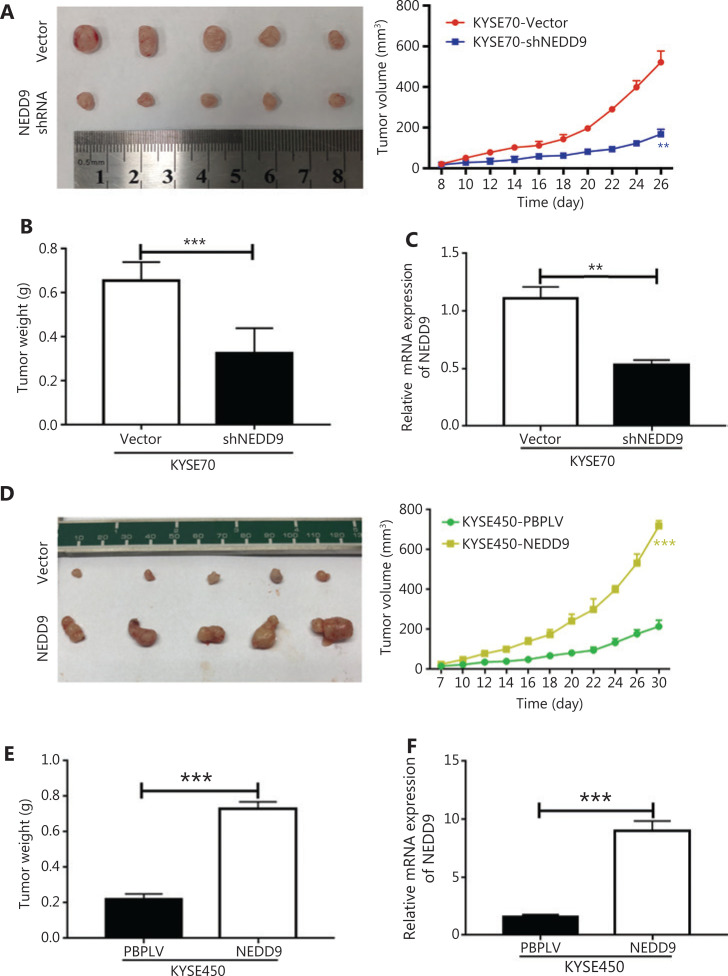
NEDD9 promotes tumorigenic potential *in vivo*. Kinetics of tumor growth *in vivo* (5 mice in each group). (A, D) Tumor volume was measured every second day and expressed in mm^3^. (B, E) Weights of xenograft tumors were evaluated. (C, F) Expression of NEDD9 was confirmed. **P < 0.01; ***P < 0.001

The most important property of CSCs is their potent tumor propagation ability. To determine whether NEDD9 was required to maintain tumorigenic potential, we examined the effects of NEDD9 disruption and overexpression on tumor formation. We found that animals bearing shNEDD9 cells showed significantly delayed tumor progression relative to the control group bearing mock vector cells (**Figure 3A–3B**). The NEDD9 expression level was significantly decreased in shNEDD9 tumors (**Figure 3C**). In contrast, NEDD9 overexpression promoted tumor growth (**Figure 3D–3F**). These data showed that NEDD9 was required to maintain the tumorigenic capacity of ESCC cells *in vivo*.

### NEDD9 regulated CXCL8 expression *via* the ERK pathway

**Figure 4:**
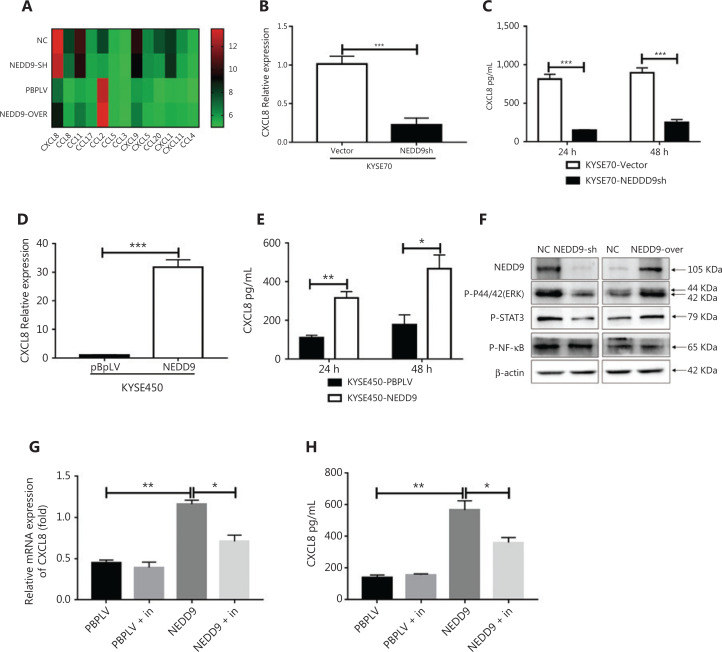
NEDD9 regulates CXCL8 expression by the ERK pathway. (A) Chemokine profiles were determined in the supernatants of NEDD9 knockdown and overexpression cells using the Human Multi-Cytokine assay. Heat map showing differential expression of genes between NEDD9 knockdown/overexpression and vector cells. The mRNA and protein expressions of CXCL8 were measured between NEDD9 knockdown and vector cells (B, C) or between NEDD9 overexpression and vector cells (D, E). (F) Activities of p-ERK1/2 (Thr202/Tyr204, 1:1,000), p-STAT3 (Tyr705, 1:1,000), and p-NF-κB (Ser536, 1:1,000) were measured by Western blot in NEDD9 knockdown and overexpression cells. (G, H) Expression of CXCL8 was determined between NEDD9 overexpression and vector cells with or without the ERK inhibitor (SCH772984). *P < 0.05; **P < 0.01; ***P < 0.001

The tumor microenvironment is the primary area where tumor cells and the host immune system interact^23^. Chemokines play a very important role in the complex cross-talk between tumor cells and the tumor microenvironment. Different immune cell subsets are recruited to the tumor microenvironment *via* interactions between chemokines and their receptors, and these populations have distinct effects on tumor progression and therapeutic outcomes^24^. Based on this information, we hypothesized that ESCC cells expressing NEDD9 communicated with the tumor microenvironment *via* chemokines. To test this hypothesis, chemokine expression was screened in ESCC cells with NEDD9 overexpression or knockdown (**Figure 4A**). We screened a selected group of chemokines (CCL2, CCL4, CCL5, CCL8, CCL11, CCL20, CCL21, CCL22, IL6, IL10, CXCL1, CXCL3, CXCL5, CXCL7, CXCL8, CXCL10, and CXCL12) using the Human Multi-Cytokine kit. Gene expression analyses revealed that only CXCL8 was significantly decreased in ESCC cells with NEDD9 knockdown, and increased in ESCC cells with NEDD9 overexpression. Next, we confirmed CXCL8 expression at the mRNA and protein levels by qRT-PCR and ELISAs (**Figure 4B–4E**). To further investigate the mechanism underlying NEDD9 regulation of CXCL8 expression in ESCC cells, NEDD9 overexpression and knockdown cell lines were used. Extracellular signal-regulated kinase (ERK), signal transducer and activator of transcription 3 (STAT3), and nuclear factor κB (NF-κB) signaling were previously reported to be involved in regulating CXCL8 expression^25^. We found that only ERK signaling was activated in NEDD9-overexpressing cells and inhibited in NEDD9-knockdown cells (**Figure 4F**). Furthermore, an ERK inhibitor (SCH772984) blocked NEDD9-induced CXCL8 expression (**Figure 4G and 4H**).

### ESCC cells with NEDD9 expression recruited G-MDSCs through CXCL8

**Figure 5:**
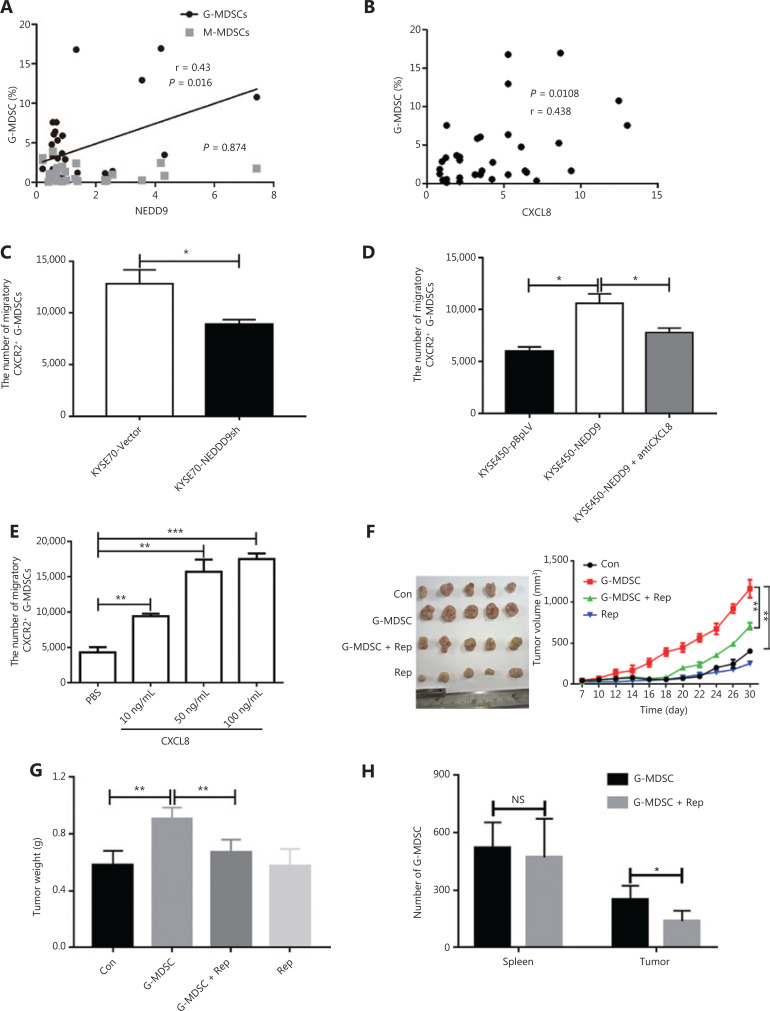
Esophageal squamous cell carcinoma (ESCC) cells with NEDD9 overexpression recruits G-MDSCs through CXCL8. (A) Correlation between myeloid-derived suppressor cells (MDSCs) and NEDD9 expression in patients with ESCC. (B) Correlation between G-MDSCs and CXCL8 expression in patients with ESCC. (C) A total of 2 × 10^5^ tumor-infiltrating G-MDSCs from patients with ESCC were seeded into the upper chamber of Transwell inserts, and the numbers of cells that migrated into the lower chamber filled with supernatant from KYSE70-Vector and KYSE70-NEDD9shRNA cells were determined by flow cytometry. (D) Numbers of cells that migrated into the lower chamber filled with supernatant from KYSE450-Vector (KYSE450-pBpLV) and KYSE450-NEDD9 cells were determined by flow cytometry. The lower chamber was filled with supernatant from KYSE450-NEDD9 in the presence of anti-CXCL8-neutralizing antibody or an isotype-matched IgG. (E) Numbers of G-MDSCs that migrated into the lower chamber filled with different concentrations of CXCL8 were counted. (F–G) Kinetics of tumor growth *in vivo* (5 mice in each group). Tumor volume was measured every second day and tumor weight was evaluated. (H) Accumulation of transferred G-MDSCs in tumor-bearing mice treated with Reparixin, measured by flow cytometry. *P < 0.05; **P < 0.01; ***P < 0.001.

CXCL8 has been associated with tumor size, depth of infiltration, and increased stage of disease in 10 different cancer types^26^. CXCL8 derived from tumors contributes to the chemotactic recruitment of G-MDSCs and to their functional control^7,27,28^. We predicted that ESCC cells expressing NEDD9 recruited MDSCs through CXCL8. Therefore, we investigated the correlation between NEDD9 expression, CXCL8 expression, and the percentage of MDSCs in ESCC tissues. The results showed that expressions of NEDD9 and CXCL8 were positively correlated with the percentage of G-MDSCs (**Figure 5A–5B**). To further characterize the relationship between ESCC cells expressing NEDD9 and G-MDSC recruitment in patients with ESCC, we established a co-culture system with Transwell membranes (4-µm) *in vitro*. The supernatants of the ESCC cell line KYSE70-vector or KYSE70-NEDD9sh cells were added to the lower chamber, and G-MDSCs were added to the upper chamber. After 24 h of incubation, the migration of G-MDSCs was significantly decreased in the KYSE70-shNEDD9 cell supernatant compared to that in the KYSE70-vector cell supernatant (**Figure 5C**). In agreement with these results, the migration of G-MDSCs was significantly increased in the supernatants of KYSE450 cells overexpressing NEDD9, when compared to that in the KYSE450-vector cell supernatants (**Figure 5D**). Furthermore, in the Transwell migration assay, we showed that G-MDSCs were attracted by recombinant CXCL8 in a dose-dependent manner (**Figure 5E**). Importantly, treatment with a neutralizing anti-CXCL8 monoclonal antibody showed reduced migration induced by the KYSE450-NEDD9 cell supernatant (**Figure 5D**). Collectively, our data demonstrated that CXCL8 mediated the recruitment of G-MDSCs induced by NEDD9 *in vitro*. We next confirmed whether CXCL8-mediated chemoattraction of G-MDSCs occurred *in vivo*. Reparixin is a pharmacological inhibitor of CXCR1 and CXCR2, which are receptors for CXCL8^29^. We found that the percentage of recruited G-MDSCs into the tumor microenvironment was positively correlated with the size and weight of the tumor (**Figure 5F and 5G**). Injection of reparixin into mice abrogated the attraction of G-MDSCs to the tumor microenvironment, but not the spleen (**Figure 5H**). These data suggested that CXCL8-mediated chemoattraction of G-MDSCs occurred *in vitro* and *in vivo*, and pharmacological inhibition of CXCR1 and CXCR2 neutralized these effects.

### G-MDSCs promoted NEDD9 expression through the NOTCH pathway in ESCC cells

**Figure 6:**
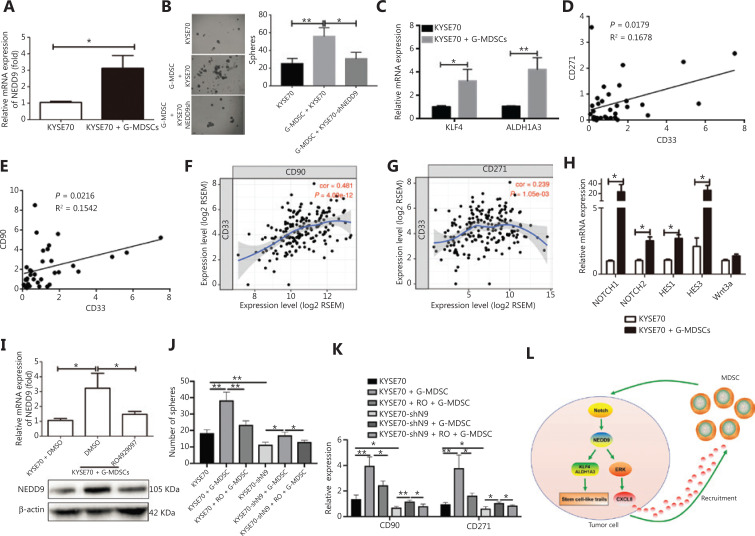
G-MDSCs promote NEDD9 expression through the NOTCH pathway in esophageal squamous cell carcinoma (ESCC) cells. (A) Expression of NEDD9 was analyzed in KYSE70 before or after co-incubation with G-MDSCs. (B) Capability of tumor sphere formation was measured in ESCC cells with NEDD9 knockdown co-cultured with G-MDSCs. Scale bar = 100 μm. (C) Expressions of KLF4 and ALDH1A3 were analyzed in KYSE70 before or after co-incubation with G-MDSCs. (D–E) Correlations between CD33 and CD271/CD90 expression in 40 ESCC tissues were evaluated by Spearman’s correlation method. (F–G) Correlations between CD33 and CD271/CD90 expression were evaluated in The Cancer Genome Atlas (TCGA; https://cancergenome.nih.gov/) database. Expression of CD33, CD271, and CD90 were obtained from TCGA . All cases were patients with ESCC without any treatment. The correlations in CD33, CD271, and CD90 expression levels were determined by Pearson’s correlation coefficients. (H) Genes in the NOTCH and WNT pathways were analyzed by real-time PCR. (I) Cells were treated with or without a NOTCH inhibitor (RO4929097) and then expression of NEDD9 was detected with or without co-incubation with G-MDSCs. (J) Cells were treated with or without NOTCH inhibitor. The capability of tumor sphere formation was measured with or without co-incubation with G-MDSCs. (K) Cells were treated with or without NOTCH inhibitor (RO4929097). Expression of stemness-related transcription factors CD271 and CD90 was detected. (L) Schematic representation of the working model of the study. *P < 0.05; **P < 0.01.

MDSCs are important immune components in the tumor microenvironment, and are thought to mediate immune suppression in patients with cancer^30,31^. MDSCs provide extrinsic signals for CSC renewal and promote tumor metastatic and tumorigenic potentials^10^. Based on the results showing that NEDD9 was required to maintain the stem-like malignant properties of ESCC cells, we predicted that MDSCs affected CSC biological behavior through NEDD9. By co-culturing ESCC cells with G-MDSC, we observed enhanced NEDD9 expression (**Figure 6A**), tumor sphere formation (**Figure 6B**), and expression of stemness-related transcription factors, KLF4 and ALDH1A3 (**Figure 6C**). Consistent with these results, NEDD9 knockdown blocked G-MDSC-induced tumor sphere formation (**Figure 6B**). G-MDSCs were associated with the levels of CD271^+^/CD90^+^ ESCC CSCs in ESCC tissues (**Figure 6D and 6E**), and we further confirmed this correlation in The Cancer Genome Atlas database (**Figure 6F and 6G**). Overall, these results suggested that G-MDSCs promoted ESCC stemness *via* NEDD9.

NOTCH and Wnt activation are observed in various cancers, and these molecules are known to control cancer progression and metastasis^32–34^. We found that the NOTCH pathway, but not the Wnt pathway, was activated in ESCC cells by G-MDSCs, as shown by the high expression of NOTCH1, NOTCH2, HES1, and HES2 transcripts (**Figure 6H**). We hypothesized that NOTCH activation was involved in G-MDSC-stimulated cancer stemness through NEDD9. To confirm this hypothesis, we blocked NOTCH signaling in the human G-MDSC and ESCC co-culture system. A NOTCH inhibitor (RO4929097) partially reduced NEDD9 expression (**Figure 6I**), tumor sphere formation (**Figure 6J**), and stemness-related transcription factors (**Figure 6K**) induced by G-MDSCs. When both NEDD9 and NOTCH signaling were inhibited, the expressions of CD90 and CD271 and tumor sphere formation induced by G-MDSCs were further abolished. These results suggested that G-MDSCs activated NEDD9 through NOTCH signaling, and induced human ESCC CSCs-like properties.

## Discussion

Most patients with ESCC are diagnosed at an advanced stage, and the overall 5-year survival is only 17%. Thus, we investigated underlying biomarkers to improve the outcomes of patients with ESCC. CSCs are thought to contribute to tumor progression, metastasis, and therapeutic resistance^30^. Identification of CSCs is crucial for advancing the understanding of ESCC biology and developing therapies.

Previous studies reported that SP cells, marked by Hoechst 33342 dye and tumor sphere-forming cells, have cancer stem-like properties^6,35,36^. We first sorted SP and non-SP cells from ESCC cell lines. Using mRNA microarray analysis, we identified 10 genes (*NEDD9*, *TM4SF1*, *IL6*, *TIPARP*, *JMJD1C*, *SFRS3*, *IER3*, *ZNF57*, *ZNF627*, and *DUSP10*) commonly upregulated in all 3 ESCC SP cells, when compared to in their respective non-SP cells. We then confirmed the expression of these 10 genes in tumor sphere-forming stem-like cells and found that only *NEDD9* was upregulated in these cells compared to in parent cells. Therefore, we focused on the role of NEDD9 in ESCC tumorigenesis and development.

NEDD9, also known as HEF1 (human enhancer of filamentation 1) or Cas-L, is a member of the multiple docking protein Cas (Crk-associated substrate) family, which has been implicated as a signaling mediator family acting on diverse biological events, including cell attachment, motility, cell cycle, apoptosis, anoikis, and tumorigenesis^37,38^. In several types of human cancers including melanoma, glioblastoma, and lung cancer, NEDD9 overexpression was positively correlated with advanced stage disease and was reported to be a poor prognostic factor^39–42^. Exogenous overexpression of NEDD9 was reported to promote the epithelial-to-mesenchymal transition and enhance cellular migration and invasion, and was suggested as a candidate biomarker of tumor aggressiveness in human malignancies^43–45^. Izumchenko et al.^46^ provided *in vivo* evidence that NEDD9 supported the activation of oncogenic signaling pathways including AKT, ERK, and SRC in breast cancer development. To date, the molecular functions of NEDD9 remain unclear in ESCCs. Here, we showed that NEDD9 was upregulated in ESCC specimens, and overall survival was significantly shorter in patients with NEDD9 overexpression. These data suggested that the NEDD9-mediated signaling pathway was dominant in the tumorigenesis and progression of ESCC.

CSCs are thought to be responsible for tumor initiation, metastasis, and recurrence^4^. We predicted that NEDD9 plays an oncogenic role by regulating the stemness of ESCC cells. To this end, we established NEDD9 overexpression and knockdown cell lines. The results showed that NEDD9 knockdown significantly inhibited the tumor sphere formation capability, which is an *in vitro* characteristic of self-renewal and proliferation capacities, stemness-related transcription factors, and CSC marker expression. Moreover, NEDD9 knockdown reduced the tumor incidence and delayed tumor progression *in vivo*. Consistently, NEDD9 overexpression had the opposite effects. These data indicated that NEDD9 was involved in maintaining the stem-like malignant properties of ESCC cells.

The tumor microenvironment is the main battle ground between tumor cells and the host immune system^47^. It has been shown that metastatic traits are acquired through interactions between tumor cells and environmental signals within the tumor microenvironment, in which MDSCs act as a major immune-suppressive component^48,49^. Data indicate that the accumulation of MDSCs is correlated with a worse clinical outcome for multiple cancer types^50^. MDSCs convey stem-like qualities similar to those of breast cancer cells, thereby enabling immune suppression and escape^10^. We hypothesized that MDSCs affected the CSC biological behavior of ESCC through NEDD9. Thus, we examined the relationship between NEDD9 expression and the percentage of MDSCs in ESCC tissues. We found that NEDD9 expression was positively correlated with G-MDSCs, which promoted the stem-like properties of ESCC cells through the NEDD9-NOTCH pathway.

Increasing evidence has supported the important roles of chemokines and their receptors in colonic inflammation and cancers^51,52^. In the tumor microenvironment, chemokines can be expressed by tumor and other cells, including immune and stromal cells. In response to specific chemokines, different immune cell subsets migrate into the tumor microenvironment and regulate tumor immune responses in a spatiotemporal manner^24^. Monocytic MDSCs can be recruited into the tumor microenvironment by CCL2^50^. CXCL1 and CXCL8 regulate G-MDSC migration and degranulation *via* CXCR1 and CXCR2 signaling^7,27,53^. In the present study, we showed that NEDD9 regulated CXCL8 expression *via* the ERK pathway in ESCC cells. Consistent with previous studies, we found that ESCC cells overexpressing NEDD9 promoted G-MDSC recruitment through CXCL8, and pharmacological inhibition of CXCR1 and CXCR2 neutralized these effects.

Several limitations of this study must be carefully considered. First, clinical investigations in our study were retrospective and conducted at a single institution. Thus, we intend to confirm our clinical findings in a prospective multi-institutional setting. Another limitation was that our experiments were performed in the context of a subcutaneous inoculation xenograft model. In the future, we will use spontaneous tumor models for further study of specific mechanisms. Furthermore, we used CD33 to identify human MDSC, but CD33-positive cells may not always display MDSC. Therefore, more appropriate markers need to be identified to clarify the phenotypic characteristics of human MDSC.

## Conclusions

In summary, our study demonstrated that NEDD9 was required for maintenance of the stem-like phenotype of ESCC. Furthermore, our results indicated that G-MDSCs activated NEDD9 through NOTCH signaling, inducing stem-like properties of human ESCC, and ESCC cells overexpressing NEDD9 promoted G-MDSC recruitment through CXCL8. Thus, anti-cancer therapy should simultaneously target host MDSCs and CSCs to improve therapeutic efficiencies.
