# Supplementary material for: NEDD9 promotes cancer stemness by recruiting myeloid-derived suppressor cells via CXCL8 in esophageal squamous cell carcinoma
Source: Cancer Biol Med. 2021 Aug 15;18(3):705–20. doi: 10.20892/j.issn.2095-3941.2020.0290 (PMC8330544; doi:10.20892/j.issn.2095-3941.2020.0290)
Supplement: Supplementary file 1 [file cbm-18-705-s001.pdf]

Supplementary materials

Table S1 Characteristics of patients with esophageal squamous cell carcinomas

Clinicopathological variables	Total cases (n = 135)
Group	
Normal tissue	90
Carcinoma tissue	135
Age (years)	
≤ 60	55
> 60	80
Gender	
Male	84
Female	51
Differentiation	
Well	45
Moderate	68
Poor	22
T stage	
1	12
2	46
3	77
Lymph node metastasis	
N0 (No)	98
N1 (Yes)	37
TNM stage	
I	10
II	96
III	29

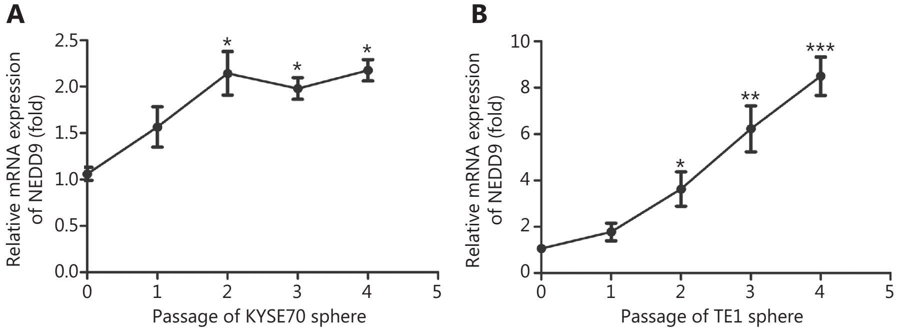

Figure S1 Expression analysis of NEDD9 in sphere-forming cells compared to in parental KESE70 (A) and TE1 (B) cells. A total of 1,000 cells were seeded in 24-well Ultra Low Attachment Plates (Corning, Corning, NY, USA) in serum-free DMEM/F12 medium supplemented with heparin, B27, EGF, and bFGF. After culturing for 7 days, the number of spheres was counted using a microscope (Leica, Wetzlar, Germany). For secondary and tertiary sphere formation, the spheres were collected, dissociated into single cells, and seeded into 24-well Ultra Low Attachment Plates. Three independent experiments were conducted in parallel. 0: parental adherent cells; 1: first passage of spheres; 2: second passage of spheres; 3: third passage of spheres; 4: fourth passage of spheres. $^{*}P < 0.05$; $^{**}P < 0.01$; $^{***}P < 0.001$ compared to the parental adherent cells.

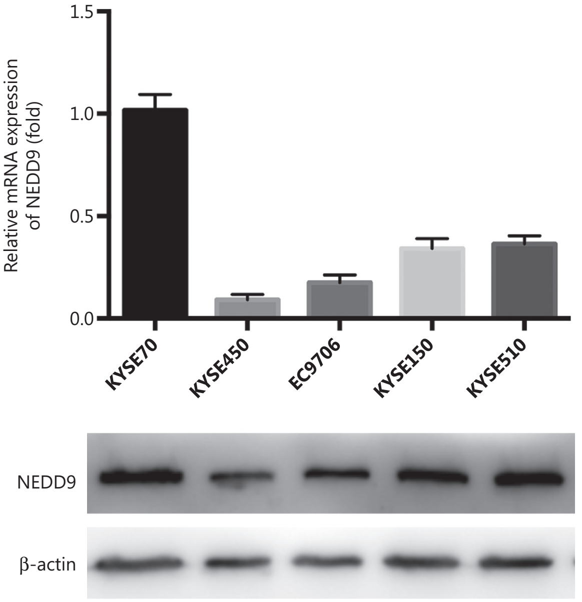

Figure S2 The mRNA and protein expressions of NEDD9 in human esophageal epithelial cell line, Het-1a, and esophageal squamous cell carcinoma cell lines were determined by real-time PCR and Western blot.
